# Re-evaluation of the significance of penicillin binding protein 3 in the susceptibility of *Listeria monocytogenes *to β-lactam antibiotics

**DOI:** 10.1186/1471-2180-12-57

**Published:** 2012-04-18

**Authors:** Agata Krawczyk-Balska, Magdalena Popowska, Zdzislaw Markiewicz

**Affiliations:** 1 Department of Applied Microbiology, Institute of Microbiology, University of Warsaw, Miecznikowa 1, 02-096 Warsaw, Poland

**Keywords:** Penicillin binding proteins, *Listeria monocytogenes*, Susceptibility to β-lactams

## Abstract

**Background:**

Penicillin binding protein 3 (PBP3) of *L. monocytogenes *has long been thought of as the primary lethal target for β-lactam antibiotics due to the excellent correlation between the MICs of different β-lactams and their affinity for this protein. The gene encoding PBP3 has not yet been directly identified in this gram-positive bacterium, but based on *in silico *analysis, this protein is likely to be encoded by *lmo1438*. However, studies examining the effects of mutations in genes encoding known and putative *L. monocytogenes *PBPs have demonstrated that inactivation of *lmo1438 *does not affect sensitivity to β-lactams.

**Results:**

In this study, overexpression of *lmo1438 *was achieved using an inducible (nisin-controlled) expression system. This permitted the direct demonstration that *lmo1438 *encodes PBP3. PBP3 overexpression was accompanied by slightly elevated PBP4 expression. The recombinant strain overexpressing PBP3 displayed significant growth retardation and greatly reduced cell length in the stationary phase of growth in culture. In antibiotic susceptibility assays, the strain overexpressing PBP3 displayed increased sensitivity to subinhibitory concentrations of several β-lactams and decreased survival in the presence of a lethal dose of penicillin G. However, the MIC values of the tested β-lactams for this recombinant strain were unchanged compared to the parent strain.

**Conclusions:**

The present study allows a reevaluation of the importance of PBP3 in the susceptibility of *L. monocytogenes *to β-lactams. It is clear that PBP3 is not the primary lethal target for β-lactams, since neither the absence nor an excess of this protein affect the susceptibility of *L. monocytogenes *to these antibiotics. The elevated level of PBP4 expression observed in the recombinant strain overexpressing PBP3 demonstrates that the composition of the *L. monocytogenes *cell wall is subject to tight regulation. The observed changes in the morphology of stationary phase cells in response to PBP3 overexpression suggests the involvement of this protein in cell division during this phase of growth.

## Background

*Listeria monocytogenes *is a ubiquitous gram-positive opportunistic pathogen that can cause very serious food-borne infections in humans, with symptoms including meningitis, frequently accompanied by septicemia and meningoencephalitis, which are particularly severe for newborns and immunocompromised individuals [1]. The antibiotics of choice in the treatment of listeriosis are the β-lactams penicillin G or ampicillin, alone or in combination with an aminoglycoside [2]. The classical target enzymes for β-lactam antibiotics are the penicillin binding proteins (PBPs). In *L. monocytogenes*, five PBPs were initially identified using radiolabeled β-lactams [3], and among these, PBP3 was thought to be the primary lethal target due to the observed low affinity of β-lactams for this protein and excellent correlation between the MICs of different β-lactams and their affinity for this protein [4-6]. Further evidence that PBP3 is the primary target for active β-lactams is that only this PBP appears to be identical in all *Listeria *spp., and blockage of this protein has lethal consequences for the bacterial cell [7].

Recent *in silico *analysis of the *L. monocytogenes *EGD genome revealed the presence of 10 genes encoding putative penicillin binding proteins and subsequently nine of these were positively verified as PBPs by the binding of a fluorescent β-lactam derivative [8,9]. Based on simple analysis of the predicted masses of these proteins, the gene likely to encode PBP3 was identified as *lmo1438 *[9]. Elegant studies by C. Hill's group on the effect of mutations in 6 of the genes encoding PBPs (including *lmo1438*) on the susceptibility of *L. monocytogenes *to β-lactams, revealed that *lmo0441 *and *lmo2229 *(PBP4) contribute to the β-lactam resistance of *L. monocytogenes*, but inactivation of *lmo1438 *did not result in obvious changes to either the sensitivity to β-lactams or the cell morphology [8].

Taking into account the seemingly contradictory nature of the aforementioned reports and the fact that the gene encoding PBP3 has yet to be directly identified, plus the absence of reports regarding the physiological function of this protein, our study focused on gene *lmo1438 *(potentially encoding PBP3). Here we describe the use of the lactococcal nisin-controlled expression (NICE) system [10] for the overexpression of this gene. This strategy was chosen because in a recently described analysis, mutational inactivation of *lmo1438 *had no obvious physiological effect [8]. In the present study, it has been directly demonstrated that *lmo1438 *encodes *L. monocytogenes *PBP3. Overexpression of this protein, which was accompanied by a slight increase in PBP4 expression, resulted in growth retardation, shortening of cells in the stationary phase of growth and minor changes in the susceptibility of *L. monocytogenes *to β-lactams. The observed changes in cell morphology indicate the involvement of PBP3 in cell division. These novel data on the overexpression of gene *lmo1438 *provide a more comprehensive view of the physiological function of PBP3 and its significance in the susceptibility of *L. monocytogenes *to β-lactams. These findings also further our understanding of the mechanisms of *L. monocytogenes *susceptibility to β-lactams, which is of direct relevance to its antibiotic resistance, the use of antibiotic therapy to treat listeriosis, as well as the ability of this bacterium to form biofilms [2,11].

## Results and discussion

### Construction of plasmid pAKB carrying the nisin-controlled expression (NICE) system and its application in *L. monocytogenes*

Given the contradictory reports on the significance of PBP3 in the susceptibility of *L. monocytogenes *to β-lactam antibiotics, it was decided to study the effects of overexpression of *L. monocytogenes *gene *lmo1438*. The lactococcal NICE system [10] was chosen for overexpression studies since it has previously been successfully used in a number of gram-positive genera, including *L. monocytogenes *[12-15]. This system consists of a two-component signal transduction system NisRK, which senses the presence of nisin and induces transcription from the promoter P_nis_. Recently developed strategies for using the NICE system either place the *nisRK *genes on the host chromosome, which allows the use of a single-plasmid system with a *nisA *promoter [13], or place both *nisRK *and the *nisA *promoter on one plasmid [16]. The first strategy was successfully used in *L. monocytogenes *by Cotter et al. [15], but here, the second approach was chosen since it is easier to use this system in various *L. monocytogenes *strains without the need for additional genetic manipulations to introduce the *nisRK *genes into the chromosome of each strain. Plasmid pAKB, a derivative of plasmid pNZ8048 carrying the *nisA *promoter, was constructed for the planned overexpression experiments. To construct this plasmid, a cassette comprised of the *nisRK *genes cloned downstream of the *L. monocytogenes hly *promoter was introduced into pNZ8048 to ensure efficient expression of these genes in *L. monocytogenes *[15]. The *lmo1438 *gene was then cloned downstream of the P_nis _promoter in pAKB to produce plasmid pAKB-*lmo1438*.

Before starting the experiments on overexpression of the *lmo1438 *gene, the susceptibility of *L. monocytogenes *to nisin was examined, since nisin is an inducer of the NICE system but it can affect or inhibit the growth of *L. monocytogenes *when used at high concentrations. The level of nisin required to completely inhibit the growth of *L. monocytogenes *EGD and of *L. monocytogenes *carrying the pAKB plasmid lacking an insert (used as a negative control in subsequent experiments) was over ten times higher than the concentration used previously to induce the NICE system in *L. monocytogenes *[15]. Furthermore, growth curves were plotted for *L. monocytogenes *pAKB grown in the presence of different concentrations of nisin as well as in the absence of this inducer to determine the concentration of nisin that has no effect on growth. These preliminary experiments showed that 15 μg/ml was the maximum concentration of nisin that did not cause any changes in the growth rate of the control strain. At higher nisin concentrations, including that used previously (45 μg/ml) to induce NICE in *L. monocytogenes *[15], a slight reduction in the growth rate of *L. monocytogenes *pAKB was observed during the exponential phase, compared to growth in the absence of nisin. The differences between the optimal nisin concentrations for growth and induction determined here and those established by Cotter et al. [15] may be due to the differential susceptibility of the strains EGD and LO28 to this peptide.

**Figure 1:**
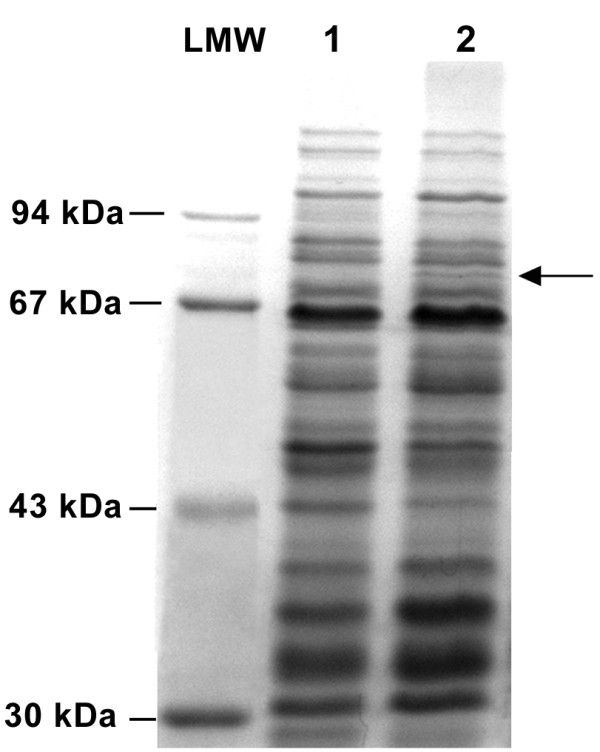
**Overexpression of the *lmo1438 *gene in *L. monocytogenes***. Membrane proteins were isolated from *L. monocytogenes *pAKB (lane 1) and *L. monocytogenes *pAKB-*lmo1438 *(lane 2) that had been grown at 37°C to exponential phase in BHI broth supplemented with nisin to a final concentration of 15 μg/ml. Proteins were separated by SDS-PAGE and stained with Coomassie brilliant blue. LMW - Protein Molecular Weight Marker. The position of the band corresponding to Lmo1438 is indicated by an arrow.

To confirm that nisin induced overexpression of the *lmo1438 *gene in *L. monocytogenes *pAKB-*lmo1438*, the cell membrane proteins of this strain and the control strain were analyzed. SDS-PAGE of isolated membrane proteins revealed the presence of an additional protein in *L. monocytogenes *pAKB-*lmo1438 *grown in the presence of 15 μg/ml nisin (Figure 1). The estimated mass of this additional protein was approximately 80 kDa, which corresponds to the predicted mass of Lmo1438 (79.9 kDa). The additional protein was detected at both 2 and 24 h following induction, but it was not observed when *L. monocytogenes *pAKB-*lmo1438 *was grown in the absence of nisin (data not shown).

To examine whether higher levels of Lmo1438 production could be achieved by changing the conditions of nisin induction and/or culture growth, *L. monocytogenes *pAKB-*lmo1438 *was grown with increasing concentrations of nisin (i.e. 30 μg/ml and 45 μg/ml), and in medium supplemented with activated charcoal, which positively regulates the *hly *promoter driving the transcription of *nisRK *[17]. In spite of these changes in the induction conditions (tested alone and in combination), no increase in the level of Lmo1438 production was observed. Since an increase in nisin concentration above 15 μg/ml had no further effect on Lmo1438 production in *L. monocytogenes *pAKB-*lmo1438*, and this concentration did not affect the growth of control strain *L. monocytogenes *pAKB, it was decided to use 15 μg/ml nisin in all subsequent physiological studies.

### Analysis of PBPs of the *L. monocytogenes *strain overexpressing gene *lmo1438*

**Figure 2:**
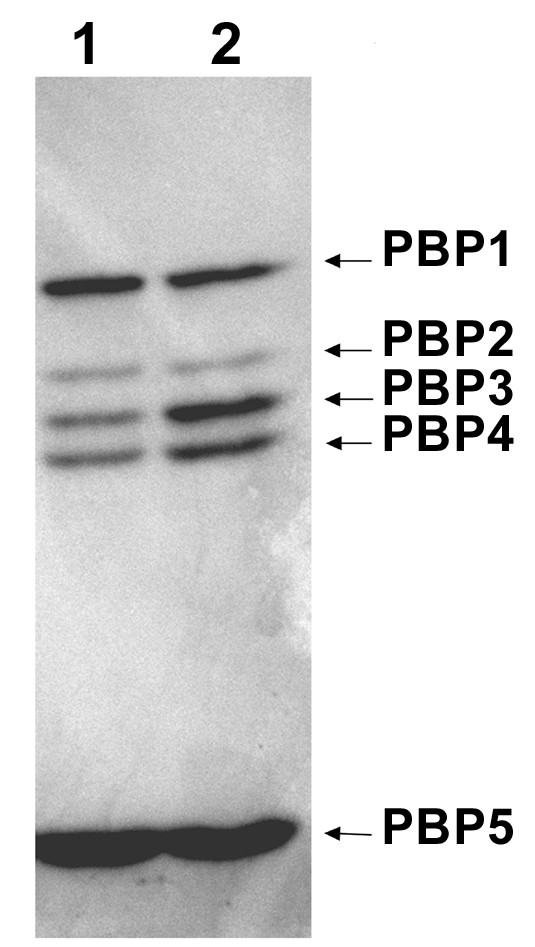
**Pattern of PBPs in *L. monocytogenes *strain overexpressing *lmo1438***. Membrane proteins (200 μg of total protein) of *L. monocytogenes *pAKB (lane 1) and *L. monocytogenes *pAKB-*lmo1438 *(lane 2) were incubated with [^3^H]benzylpenicillin at a saturating concentration of 5 μg/ml and the radiolabeled PBPs were separated by SDS-PAGE and detected by fluorography. The PBP corresponding to each band is indicated on the right.

To determine whether *lmo1438 *encodes PBP3, membrane proteins of *L. monocytogenes *pAKB and *L. monocytogenes *pAKB-*lmo1438 *were incubated with [^3^H]benzylpenicillin, then separated by SDS-PAGE followed by fluorography to detect the labeled PBPs. This assay clearly demonstrated an increased level of PBP3 in *L. monocytogenes *pAKB-*lmo1438 *(Figure 2). Densitometric analysis of PBPs produced by both strains revealed that the amount of PBP3 in *L. monocytogenes *pAKB-*lmo1438 *was 3.5-fold greater than in *L. monocytogenes *pAKB (Table 1). This result proved that *L. monocytogenes *gene *lmo1438 *does indeed encode PBP3. Interestingly, *L. monocytogenes *pAKB-*lmo1438 *also showed a small but significant increase in the expression of PBP4 compared with *L. monocytogenes *pAKB. This protein, encoded by gene *lmo2229*, was previously shown to have glycosyltransferase, transpeptidase and carboxypeptidase activities [18]. The expression of PBP4 is directly regulated by the *hpk1021-rrp1022 *two-component system [19], which in turn is subject to regulation by the LisRK two-component system [15]. Both of these two-component systems play essential roles in regulating the structure of the *L. monocytogenes *cell envelope, but they are also involved in resistance to nisin, so it was unclear whether the elevated level of PBP4 observed in *L. monocytogenes *pAKB-*lmo1438 *was the consequence of nisin use or an effect of PBP3 overexpression. Therefore, an analysis of PBP proteins isolated from *L. monocytogenes *pAKB cultured with and without nisin was performed. This showed that the addition of nisin at a concentration of 15 μg/ml had no effect on the production of PBPs by the control strain (data not shown). Furthermore, the elevated level of PBP4 observed in the strain *L. monocytogenes *pAKB-*lmo1438 *compared with *L. monocytogenes *pAKB, when both were cultured in the presence of nisin, indicated that this phenomenon is a consequence of PBP3 overexpression.

**Table 1:** Relative amounts of PBPs in recombinant *L.monocytogenes* strains


**Protein**	**Amount of PBP protein *^a^***	
	
	***L. monocytogenes *pAKB**	***L. monocytogenes *pAKB-*lmo1438***

PBP1	4.48 (± 0.45)	4.21 (± 0.81)
PBP2	1*^b^*	0.96 (± 0.08)
PBP3	1.66 (± 0.15)	5.78 (± 0.47)*^c^*
PBP4	1.67 (± 0.05)	3.2 (± 0.34)*^c^*
PBP5	12.05 (± 0.42)	12.01 (± 1.03)

*^a ^*Average results of densitometric analysis of three independent fluorograms.

*^b ^*Values were normalized to the band intensity of PBP2 from *L. monocytogenes *pAKB, which was assigned the value of 1.

*^c ^*Indicates band with intensity significantly different (*P *< 0.05; acc. to Student's *t*-test) from the corresponding band of the control strain.

### Effect of PBP3 overproduction on growth and cell morphology of *L. monocytogenes*

**Figure 3:**
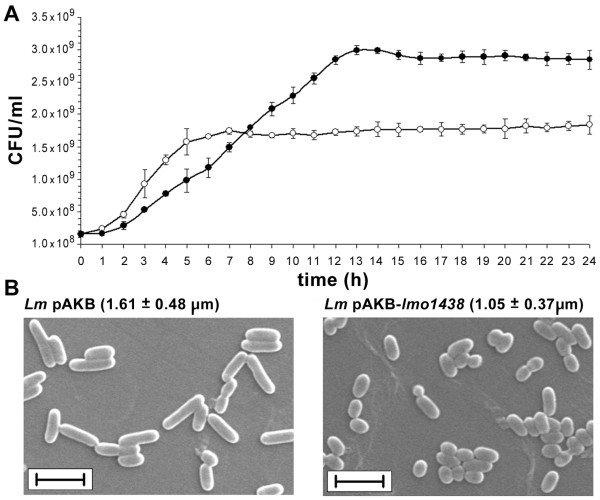
**Effect of overproduction of PBP3 on growth and morphology of *L. monocytogenes***. (**A**) Growth of *L. monocytogenes *pAKB (○) and *L. monocytogenes *pAKB-*lmo1438 *(•) incubated in BHI broth at 37°C following nisin induction, determined by serial dilution of the cultures and enumeration of viable cells on BHI agar. Error bars represent standard deviation from the means of three independent experiments, each performed in triplicate. (**B**) SEM images of *L. monocytogenes *pAKB (*Lm *pAKB) and *L. monocytogenes *pAKB-*lmo1438 *(*Lm *pAKB-*lmo1438*) cells grown overnight in BHI broth at 37°C following nisin induction. The mean cell lengths (± SD), determined by measuring 100 cells of each strain, are shown in parentheses. Bar = 2 μm.

Since mutation of the *lmo1438 *gene did not cause any changes in the growth and cell morphology of *L. monocytogenes*, the physiological role of PBP3 is unclear. To better understand the cellular function of PBP3, the effect of increased production of this protein on the growth and morphology of *L. monocytogenes *was examined. The growth rate of the strain overproducing PBP3 was visibly retarded during the exponential phase of growth, when the doubling time of *L. monocytogenes *pAKB-*lmo1438 *was 116 min compared to 62 min for *L. monocytogenes *pAKB. However, in the stationary phase of growth the culture of *L. monocytogenes *pAKB-*lmo1438 *reached a higher OD_600 _value compared to the control strain, which correlated with a significantly higher number of viable bacteria in this phase of growth (Figure 3A).

Analysis of cell morphology by scanning electron microscopy revealed that *L. monocytogenes *pAKB and *L. monocytogenes *pAKB-*lmo1438 *cells appeared similar in the exponential phase of growth and the length and diameter of cells of both strains did not differ statistically. However, an analysis of cell morphology of *L. monocytogenes *pAKB-*lmo1438 *and the control strain in the stationary phase of growth showed that the cells of both strains had the same diameter, but those of the former strain were significantly shorter (Figure 3B). The reduced growth rate of *L. monocytogenes *pAKB-*lmo1438 *cannot solely be attributed to the overexpression of PBP3, since an elevated level of PBP4 expression was also found in the recombinant strain, and disruption of the *lmo2229 *gene indicates that PBP4 is essential for the growth of *L. monocytogenes *[18]. Therefore, the observed growth retardation may be the result of the overexpression of PBP3, PBP4 or of both these proteins. The clear reduction in the cell length of *L. monocytogenes *pAKB-*lmo1438*, with no change in cell diameter, suggests a role for PBP3 in cell division. Current models of bacterial cell wall synthesis suggest that distinct wall-synthetic complexes act in an alternating fashion during the life cycle, to first drive cell elongation by the insertion of peptidoglycan into the cylindrical wall, followed by the switching of most wall-synthetic activity to septum production [20]. In *E. coli*, the genes required for septation have been identified and most are designated *fts *(filamentation, temperature sensitive), of which FtsI (a PBP with monofunctional transpeptidase activity) is a major protein of the cell division complex or divisome [21]. Bioinformatic analysis of the *L. monocytogenes *PBP3 showed that this protein could potentially act as an FtsI cell division transpeptidase [8]. We hypothesize that an excess of PBP3 disturbs the balance between the activities of the cell elongation and cell division complexes, and the majority of peptidoglycan synthesis might be carried out by the septum synthetic machinery. This would explain the production of shorter cells by *L. monocytogenes *pAKB-*lmo1438*. We assume that the formation of short cells is triggered by PBP3 overexpression, rather than increased PBP4 abundance, since transglycosylases are part of the general peptidoglycan synthetic machinery and are not specific for cell division. However, a number of less specialized enzymes are also required for lateral expansion [22].

The postulated participation of PBP3 in cell division is evidently limited to the stationary phase of growth which may result from the presence of a second protein with FtsI activity in *L. monocytogenes*. Indeed, Lmo2039 is also a potential FtsI cell division transpeptidase and it is suggested that the *lmo2039 *mutation is lethal for *L. monocytogenes *[8]. It seems therefore, that Lmo2039 is the main protein involved in division of *L. monocytogenes*. The influence of PBP3 on cell morphology is manifested in the stationary phase cells, which suggests that this protein may play a unique role during this phase of growth. This hypothesis is supported by the unchanged cell morphology of *L. monocytogenes *in the exponential phase of growth, both in the absence of PBP3 [8] and when this protein is overexpressed.

### Effect of overexpression of PBP3 on the susceptibility of *L. monocytogenes *to β-lactams

**Figure 4:**
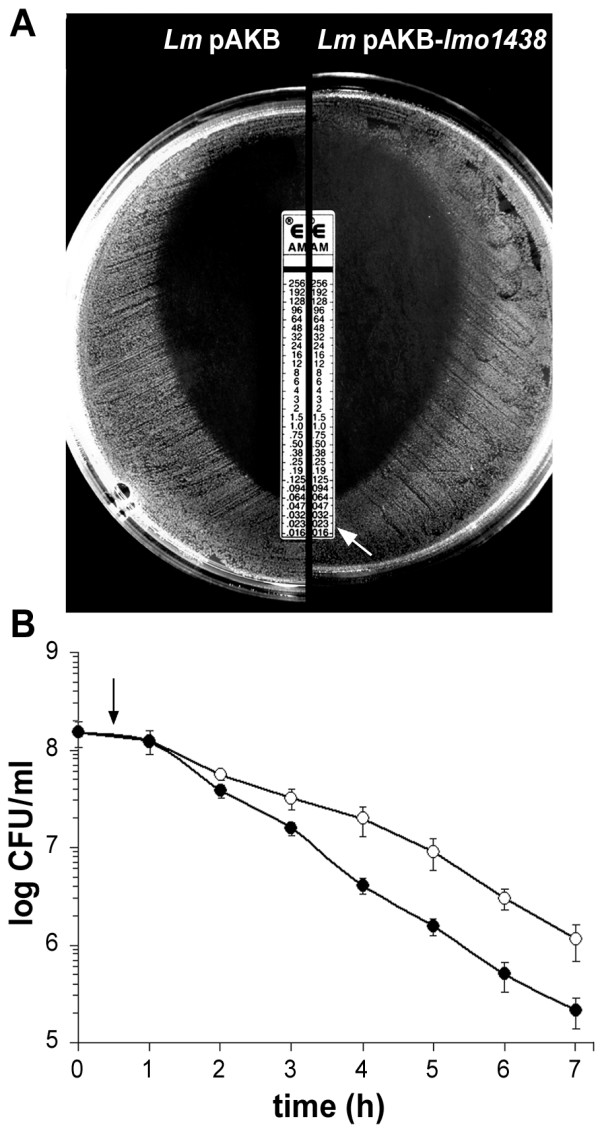
**Effect of overproduction of PBP3 on the susceptibility of *L. monocytogenes *to β-lactams**. (**A**) Susceptibility of *L. monocytogenes *pAKB (*Lm *pAKB) and *L. monocytogenes *pAKB-*lmo1438 *(*Lm *pAKB-*lmo1438*) to ampicillin measured using the E-test. The extent of the zone of partial autolysis of *L. monocytogenes *pAKB-*lmo1438 *is indicated by an arrow. (**B**) Survival of *L. monocytogenes *pAKB (**○**) and *L. monocytogenes *pAKB-*lmo1438 *(**•**) in the presence of a lethal dose of penicillin G (0.6 μg/ml). Following nisin induction, penicillin G was added (at the time indicated by an arrow) to the cultures in BHI broth and incubation at 37°C was continued. Survival was measured by performing viable cell counts. Error bars represent standard deviations from the means of three independent experiments, each performed in triplicate.

To determine whether PBP3 plays a role in β-lactam resistance, *L. monocytogenes *pAKB and *L. monocytogenes *pAKB-*lmo1438 *were tested for their susceptibility to penicillins, cephalosporins, monobactams and carbapenems using an antibiotic disk sensitivity assay. This preliminary assay did not reveal any significant changes in the sensitivity to β-lactams caused by the overproduction of PBP3 - the diameters of the zones of bacterial growth inhibition surrounding the filter disks were identical after 24 h incubation. However, after 48 h incubation, partial autolysis of the bacterial growth in the presence of subinhibitory concentrations of penicillin G, ampicillin, amoxicillin, mezlocillin and imipenem was observed (data not shown). Penicillin G, ampicillin and amoxicillin were then chosen for MIC determination using the E-test. This assay confirmed the results of the antibiotic disk tests, namely that both strains were equally susceptible to the β-lactams tested and that in the case of the strain overexpressing PBP3, a zone of partial autolysis of the bacterial lawn was observed at an antibiotic concentration three to four times lower than the MIC. The results for ampicillin are presented in Figure 4A. A survival assay was also performed for *L. monocytogenes *pAKB and *L. monocytogenes *pAKB-*lmo1438 *by culturing these strains in broth supplemented with a lethal dose of penicillin G. The optical density of the *L. monocytogenes *pAKB-*lmo1438 *culture decreased at a faster rate than that of the control strain, which correlated with the more rapid elimination of viable bacteria from the culture (Figure 4B). Keeping in mind that in the constructed strain an increased level of PBP4 expression was also observed, which was found to contribute to the susceptibility of *L. monocytogenes *to β-lactams [8,23], the changes in the susceptibility of *L. monocytogenes *pAKB-*lmo1438 *to β-lactam antibiotics may be not only an effect of PBP3 overexpression. Thus, it seems probable that the altered susceptibility of this strain to β-lactams is the effect of overexpression of PBP3, PBP4 or both of these proteins. Regardless of the reason for the altered susceptibility, it may definitely be concluded that overexpression of PBP3 (accompanied by increased levels of PBP4) leads to minor changes in the susceptibility of *L. monocytogenes *to β-lactams without any change in the MIC values. Together with the lack of changes in β-lactam MIC values in the case of the *lmo1438 *mutant strain reported by Guinane et al. [8], this result demonstrates that the role of PBP3 is non-essential in the β-lactam resistance of *L. monocytogenes*. These findings concerning the β-lactam antibiotic resistance of *L. monocytogenes *are important with regard to the treatment of listeriosis and the ability of this bacterium to form biofilms [2,11]. The latter complex issue has recently been reviewed by Renier et al. [11].

## Conclusions

The findings of the present study have helped to elucidate the somewhat conflicting results regarding the contribution of PBP3 to the β-lactam susceptibility of *L. monocytogenes*. Using the NICE expression system, it has been directly shown that PBP3 is encoded by the *lmo1438 *gene. Despite the excellent correlation between the MICs of different β-lactams and their affinity for PBP3 [4,5], neither the absence [8] nor an excess of this protein affects the susceptibility of *L. monocytogenes *to β-lactams, and so it is not the primary lethal target of these antibiotics.

An interesting additional observation was that PBP3 overexpression is accompanied by increased expression of PBP4. This finding indicates that the composition of the *L. monocytogenes *cell wall is subject to tight regulation, but it also makes it difficult to analyze the physiological role of PBP3 on the basis of overexpression studies, since the observed changes in growth rate and antibiotic sensitivity cannot be attributed to PBP3 overexpression alone. The overexpression of PBP3 induced the formation of short cells in the stationary phase of growth, which strongly suggests the involvement of PBP3 in cell division at this growth stage. It is possible that the changes in cell morphology produced by overexpression of PBP3 may be due to a putative FtsI activity, whereas the parallel increase in the expression of PBP4 (a cell wall synthetic enzyme not specific for cell division) could play an auxiliary role in this process.

Finally, in the course of clarifying the contribution of PBP3 to the β-lactam susceptibility of *L. monocytogenes*, a new vector was constructed that is suitable for the overexpression of genes of interest in *L. monocytogenes*. The placement of components of the NICE system on a single plasmid provides an easy to use tool for expressing any protein of choice in *L. monocytogenes*. The construction of the plasmid pAKB and its successful application in *L. monocytogenes *are important in view of the limited number of existing inducible gene expression systems for *Listeria *spp.

## Methods

### Bacterial strains, plasmids and growth conditions

*E. coli *DH5α, used in cloning procedures, was grown aerobically at 37°C in Luria-Bertani (LB) medium. *L. monocytogenes *EGD was kindly provided by S.J. Foster, University of Sheffield, United Kingdom. *L. monocytogenes *EGD and its derivatives were grown in Brain Heart Infusion medium (BHI, Oxoid) at 37°C. Plasmids pNZ8048 [10] and pNZ9530 [12] were a kind gift from Michiel Kleerebezem, NIZO, Ede, The Netherlands. Plasmid pUC18 [24] was obtained from the collection of the Institute of Microbiology, University of Warsaw. Ampicillin (100 μg/ml) and chloramphenicol (10 μg/ml) were added to broth or agar media as required. When necessary, solid LB medium was supplemented with 0.1 mM IPTG (isopropyl b-D-1-thiogalactopyranoside) and 20 μg/ml X-Gal (5-bromo-4-chloro-3-indolyl-b-D-galactopyranoside).

### DNA manipulations and reagents

Standard protocols were used for recombinant DNA techniques [25]. DNA fragments were isolated from agarose gels with the QIAquick Gel Extraction Kit (QIAGEN). DNA fragments from PCR and after enzymatic reactions were purified with the QIAquick PCR Purification Kit (QIAGEN). Plasmid DNA was isolated from *E. coli *with the Plasmid Miniprep Plus Kit (A&A Biotechnology). The isolation of chromosomal DNA from *L. monocytogenes *was performed as previously described [26]. Restriction enzymes, nuclease S1, T4 DNA ligase and Pfu DNA polymerase were purchased from Fermentas and used according to the manufacturer's instructions. The oligonucleotide primers used in this study are shown in Table 2.

**Table 2:** PCR primers used in this study


**Primer**	**Sequence (5'→ 3')**

HlyA^a^	GCGGGTACCAGGTAGAGCGGACATCCATTG
HlyB^b, c, d^	*GTTTTA**GGATCC**CCCGGG*GGGTTTCACTCTCCTTCTAC
HlyC^b, c^	CCCGGG**GGATCC**TAAAACCGCTTAACACACACG
HlyD^e^	GCGTCTAGATTCTTCCCCGACAGAATCTGC
NisR F	CCCACTAAACAATCGGAGG
NisK R^c^	GCG**GGATCC**CAGAAATTAAACCAAACAAAATTTTC
Oepbp3 F	CGTGAAACTAAATTTTAGAAAAAAGAAAAAAG
Oepbp3 R^f^	GCGGCATGCGATTAATTTTCGGTTTGTTCTGATTG

^a ^Nucleotide substitutions to create KpnI site are underlined

^b ^Nucleotide substitutions to create SmaI site are underlined

^c ^Nucleotide substitutions to create BamHI site are in boldface

^d ^Overhang complementary to SOE primer is in italics

^e ^Nucleotide substitutions to create XbaI site are underlined

^f ^Nucleotide substitutions to create SphI site are underlined

### Construction of plasmid pAKB carrying the nisin-controlled expression (NICE) system and its derivative pAKB-*lmo1438*

A strategy based on the amplification and cloning of PCR products was devised to construct a plasmid carrying the NICE system suitable for the overexpression of *L. monocytogenes *genes. With *L. monocytogenes *EGD genomic DNA as the template, primers HlyA and HlyB were used to amplify a fragment of approximately 0.4 kb comprising the promoter region of the *hly *gene, and primers HlyC and HlyD were used to amplify a 0.4-kb fragment comprising the terminator of this gene. These two fragments were used as the templates for splicing by overlap extension PCR. A 0.8-kb fragment, representing the region surrounding *L. monocytogenes hly*, but with the gene precisely removed, was then amplified using the flanking primers HlyA and HlyD. This DNA fragment was digested with KpnI and XbaI and cloned in vector pUC18 to produce plasmid pUC18-P*_hly. _*A fragment of approximately 2.3 kb comprising the *nisRK *operon was amplified from plasmid pNZ9530 using primers nisR F and nisK R (containing incorporated BamHI site). This fragment was digested with BamHI and cloned in plasmid pUC18-P*_hly _*that had been digested with SmaI and BamHI, which cleave the sites within primers HlyB and HlyC, respectively. Thus, the *nisRK *operon was cloned into the location formerly occupied by the *hly *gene to produce plasmid pUC18-P*_hly_*-*nisRnisK*. A DNA fragment of approximately 3.1 kb comprising the promoter region of the *hly *gene, the *nisRK *operon and the terminator of *hly *was excised from pUC18-P*_hly_*-*nisRnisK *by digestion with KpnI and XbaI, gel purified and cloned in plasmid pNZ8048 digested with the same restriction enzymes. The resulting plasmid was designated pAKB. A fragment of approx. 2.2 kb comprising the *lmo1438 *gene was amplified from *L. monocytogenes *EGD genomic DNA using primers Oepbp3 F (containing the *lmo1438 *start codon) and Oepbp3 R (containing the *lmo1438 *stop codon and a SphI site). This fragment was digested with SphI and cloned into NcoI-digested (ends blunted with nuclease S1 after digestion) and subsequently SphI-digested pAKB, to generate a transcriptional fusion between the nisin-inducible *nisA *promoter on pAKB and the *lmo1438 *gene, maintaining the original GTG start codon of *lmo1438*. The predicted sequence of this construct was confirmed by DNA sequencing. Plasmids pAKB and pAKB-*lmo1438 *were introduced into *L. monocytogenes *EGD by electroporation [27] and transformants were selected on BHI agar plates containing 10 μg/ml chloramphenicol. The obtained strains were designated *L. monocytogenes *pAKB and *L. monocytogenes *pAKB-*lmo1438*, respectively.

### Growth in the presence of nisin

*L. monocytogenes *strains were grown overnight with shaking at 37°C. The cultures were diluted 1:50 into fresh BHI medium and grown at 37°C with aeration to an optical density at 600 nm (OD_600_) of 0.2. At this point, nisin powder (containing 2.5% nisin; Sigma) was added to the cultures to produce a final nisin concentration of 15 μg/ml. The growth rates of *L. monocytogenes *pAKB and *L. monocytogenes *pAKB-*lmo1438 *were compared spectrophotometrically by recording the OD_600 _of the cultures and by determining the number of viable bacteria, following serial dilution and plating on BHI agar.

### Preparation of membrane fractions

Membrane fractions from *L. monocytogenes *strains were prepared essentially as described previously [6]. Briefly, strains were grown at 37°C to exponential phase (OD_600 _of 0.5) in 200 ml of BHI broth supplemented with nisin powder to a final concentration of 15 μg/ml. Cells were harvested by centrifugation for 10 min at 8000 × g at 4°C and washed twice in 10 ml of 20 mM phosphate buffer (pH 7.0). The pellet was resuspended in 8 ml of the same buffer supplemented with protease inhibitor PMSF (Sigma) to a final concentration of 1 mM. Glass sand (0.5 mm diameter; Sigma) was added to the suspension and the cells were disintegrated by sonication in a VCX-600 ultrasonicator (Sonics and Materials, U.S.A.) at an amplitude of 20%. Unbroken cells and glass sand were removed by low speed centrifugation and the membrane fractions in the supernatant were collected by centrifugation at 100,000 × g for 30 min at 4°C and suspended in 200 μl of 20 mM phosphate buffer (pH 7.0). The protein concentration in samples was quantified using a Bicinchoninic Acid protein assay kit (Sigma) and, where necessary, the concentration was adjusted to 10 mg/ml.

### Labeling of PBPs with radioactive benzylpenicillin

The labeling of PBPs with radioactive benzylpenicillin was carried out essentially as described previously [3]. Briefly, aliquots (20 μl) of the *L. monocytogenes *membrane suspension (10 mg of protein per ml) were incubated for 15 min at 37°C with [^3^H]benzylpenicillin (Amersham) added to a final concentration of 5 μg/ml (previously found to represent the saturating concentration). Binding was terminated by the addition of excess benzylpenicillin (final concentration 0.5 mg/ml) and the detergent sarkosyl (final concentration 2% v/v), followed by 20 min incubation at room temperature.

### Analysis of cell membrane proteins and PBPs

Sample buffer (62.5 mM Tris-HCl, 2% SDS, 10% glycerol, 0.01% bromophenol blue, 5% 2-mercaptoethanol, pH 6.8) was added to the *L. monocytogenes *cell membrane suspensions, the samples were boiled for 2 min and then subjected to sodium dodecyl sulfate - 10% polyacrylamide gel electrophoresis. In the case of unlabeled proteins, the gels were stained with Coomassie brilliant blue to visualize the protein bands. In the case of [^3^H]benzylpenicillin-labeled PBPs, the gels were processed by impregnation with an organic scintillant and fluorography was used to detect the radiolabeled PBP bands. For the visualization of fluorograms and densitometric analysis, ImageQuant™ 300 and ImageQuant™ TL software (GE Healthcare, United Kingdom) were used, respectively. The presented results are the average of data from three independent experiments.

### Scanning electron microscopy

Scanning electron microscopy was used to examine exponential and stationary phase cells of *L. monocytogenes *strains grown at 37°C in BHI medium supplemented with nisin powder to a final concentration of 15 μg/ml. Culture samples of 10 ml were harvested by centrifugation at (7000 × g, 10 min, at room temperature). The cells were fixed for 30 min in 4% paraformaldehyde, washed three times in phosphate-buffered saline (pH 7.4), then dehydrated using a graded ethanol series (25, 50, 75, 96% ethanol; 15 min for each step). One drop of cell suspension was spread on a microcover, coated with gold, and examined using a LEO 1430VP scanning electron microscope (SEM).

### Antibiotic susceptibility tests

The susceptibility of *L. monocytogenes *strains to penicillin, ampicillin and amoxicillin was determined using an E-test (AB Biodisk, Sweden). Overnight cultures of the strains were diluted into fresh BHI medium and grown at 37°C with aeration to an OD_600 _of 0.2. The cultures were diluted and a suspension containing 10^6 ^CFU/ml was swabbed onto plates of BHI agar supplemented with nisin to a final concentration of 15 μg/ml. E-test strips were placed on the inoculated plates, which were then incubated at 37°C for 24 h and 48 h before the results were recorded. Survival of the *L. monocytogenes *strains was tested in the presence of a lethal dose of penicillin G. Overnight cultures of the strains were diluted (1:50) into BHI broth and grown at 37°C to early exponential phase (OD_600 _of 0.2) before nisin powder was added to a final concentration of 15 μg/ml. The cultures were then grown for a further 30 min before penicillin G was added to a final concentration of 0.6 μg/ml. The effect of the antibiotic on the *L. monocytogenes *strains was compared spectrophotometrically by recording the OD_600 _of the cultures and by determining the number of viable bacteria, following serial dilution and plating on BHI agar.

## Authors' contributions

AK-B carried out the molecular cloning to create the constructs to apply the NICE system in *L. monocytogenes*, performed the analysis of PBPs as well as the susceptibility studies, and helped to draft the manuscript. MP carried out the studies on growth and cell morphology of the obtained recombinant strains. ZM conceived part of the study, participated in its design and coordinated the preparation of the manuscript. All authors read and approved the final version of the manuscript.
